# GelMA micropattern enhances cardiomyocyte organization, maturation, and contraction via contact guidance

**DOI:** 10.1063/5.0182585

**Published:** 2024-05-01

**Authors:** Bin Zhang, Yichen Luo, Xue Zhou, Lei Gao, Xiaohong Yin, Huayong Yang

**Affiliations:** 1 State Key Laboratory of Fluid Power and Mechatronic Systems, Zhejiang University, Hangzhou 310027, People's Republic of China; 2 School of Mechanical Engineering, Zhejiang University, Hangzhou 310027, People's Republic of China

## Abstract

Cardiac tissue engineering has emerged as a promising approach for restoring the functionality of damaged cardiac tissues following myocardial infarction. To effectively replicate the native anisotropic structure of cardiac tissues *in vitro*, this study focused on the fabrication of micropatterned gelatin methacryloyl hydrogels with varying geometric parameters. These substrates were evaluated for their ability to guide induced pluripotent stem cell-derived cardiomyocytes (CMs). The findings demonstrate that the mechanical properties of this hydrogel closely resemble those of native cardiac tissues, and it exhibits high fidelity in micropattern fabrication. Micropatterned hydrogel substrates lead to enhanced organization, maturation, and contraction of CMs. A microgroove with 20-*μ*m-width and 20-*μ*m-spacing was identified as the optimal configuration for maximizing the contact guidance effect, supported by analyses of nuclear orientation and F-actin organization. Furthermore, this specific micropattern design was found to promote CMs' maturation, as evidenced by increased expression of connexin 43 and vinculin, along with extended sarcomere length. It also enhanced CMs' contraction, resulting in larger contractile amplitudes and greater contractile motion anisotropy. In conclusion, these results underscore the significant benefits of optimizing micropatterned gelatin methacryloyl for improving CMs' organization, maturation, and contraction. This valuable insight paves the way for the development of highly organized and functionally mature cardiac tissues *in vitro*.

## I. INTRODUCTION

Cardiovascular disease is one of the leading causes of death at the global level,^1–3^ prompting the advancement of cardiac tissue engineering as a forefront area for researching and treating heart diseases.^4,5^ Myocardial infarction (MI) is one of the main causes of death in cardiovascular disease patients. In 2015, there were approximately 15.9  × 10^6^ patients with myocardial infarction worldwide.^2^ In comparison to the low cell transplantation and retention rates associated with traditional cell injections, cardiac patch offers immense potential as a treatment approach for myocardial infarction.^6,7^ Biomaterials provide an optimal microenvironment for CMs, preventing substantial cell loss and allowing the creation of biomimetic cardiac tissue.^8^ This possibility enables the creation of biomimetic cardiac tissue and its use in the repair of damaged myocardial tissue resulting from infarction.^9,10^

Cardiac tissue consists of highly aligned CMs that play key roles in myocardial functions, such as rotation and torsion.^11^ Highly organized CMs measuring approximately 150 *μ*m in length and 20 *μ*m in width^12^ have demonstrated superior contractile performance, enhanced electrical coupling, and advanced maturation compared to their randomly distributed counterparts.^13–16^ This anisotropic structure largely contributes to the directed propagation of electrical signal and contractile force.^17,18^ Inducing the alignment of CMs is currently one of the research focuses. Various micropatterns such as grooves, wells, and pillars, have been developed for constructing anisotropic cardiac tissues.^19–23^ These micropatterns are generated using nano- or microfabrication technologies, including micro-molding and additive manufacturing.^24,25^ Meanwhile, it has been suggested that 20 μm was the optimal size for guiding the alignment of CMs.^14,26–29^ One explanation is that this size resembles the average functional intercapillary distance.^13,30^ However, other geometrical parameters that might also influence the alignment of CMs are not yet thoroughly investigated. These parameters include the spacing between geometrical units and the shape of micropatterns. In addition, the relationship between organization, maturation, and contraction of CMs has not been investigated systematically. In addition, the underlying biological mechanism of contact guidance is yet to be exploited.

In this investigation, we employed microfabrication through micro-molding to generate a range of distinct micropatterns on the photocrosslinkable material known as gelatin methacryloyl (GelMA) hydrogel. GelMA is a type of synthesized gelatin-based hydrogel that is widely used in tissue engineering.^13,31–33^ It features tunable biochemical properties and excellent biocompatibility due to the presence of cell binding moieties.^28,34,35^ In addition, By controlling the grafting rate of methacrylic anhydride (MA) and adjusting the parameters during the cross-linking process, the mechanical properties of GelMA can be easily tuned within a wide range (elastic modulus from 2 to 260 kPa) to resemble that of native cardiac tissues.^11,36–38^ Therefore, GelMA is an appropriate material for constructing micropatterns that guide cell organization.

This study aims to investigate the influence of different micropattern parameters, such as width, spacing, and shape, on the organization, maturation, and contraction of iPSC-CMs. Therefore, we produced a variety of GelMA substrates with micropatterns and then seeded iPSC-CMs on them. To assess the influence of contact guidance, we examined the orientation of cell nuclei and the organization of cytoskeletal F-actin. Furthermore, we conducted a comprehensive assessment of maturation and contractility through a combination of immunofluorescence staining, RNA-sequencing, and video-based analysis of cardiac beating. Finally, we propose an enhanced micropattern configuration to enhance the organization, maturation, and contractile performance of CMs. Our results offer valuable insights for the design, modeling, and fabrication of functional cardiac tissues *in vitro*, as well as for revealing the biological process of contact guidance.

## II. RESULTS

### A. Fabrication of micropatterns

**FIG. 1.**
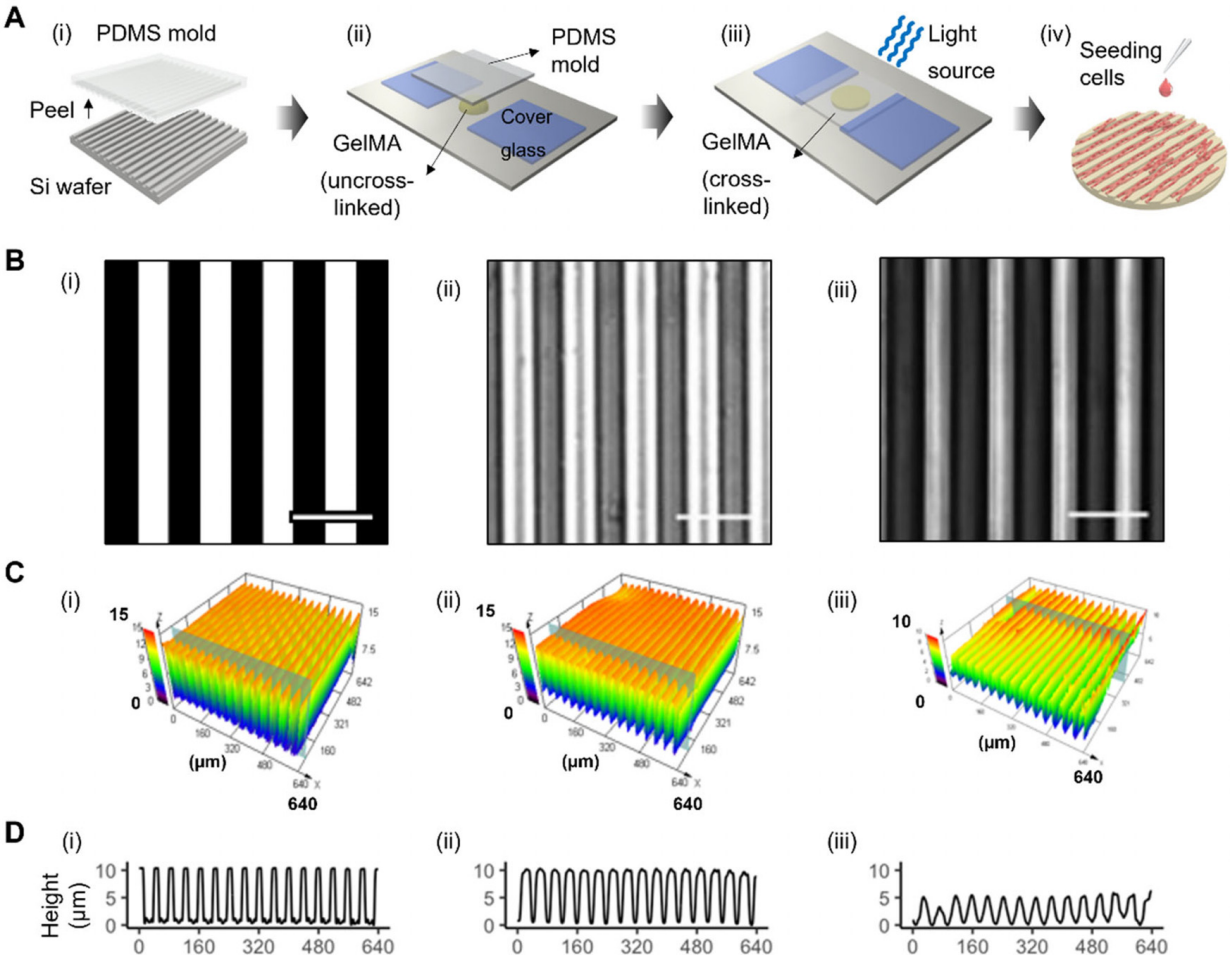
Fabrication of micropatterned GelMA and characterization of micropatterns. (a) Fabrication process of a micropatterned GelMA, (i) peel PDMS mold from micropatterned silicon wafer; (ii) dispense uncrosslinked GelMA on a substrate; (iii) cross-link GelMA using 405 nm blue light source; (iv) seed iPSC-CMs on the micropatterned GelMA. (b) Designed (i) micropattern, and their micrographs on (ii) PDMS mold and (iii) GelMA substrate. (c) Surface topography, and (d) height profile of the selected section of micropatterned (i) silicon wafer, (ii) PDMS mold, and (iii) GelMA with 20-*μ*m grooves and 20-μm spacing. Scale bar = 50 *μ*m.

The fabrication process of micropatterned GelMA substrates is shown in Fig. 1(a). Briefly, micropatterns are manufactured on silicon wafers and transferred onto the surface of GelMA by a two-step molding process using PDMS as a medium. The dual cross-linking property of GelMA enables the fast gelation by reducing temperature and the irreversible forming of micropatterns by UV exposure.^39^ Micropatterns were intactly reproduced through this fabricating process [Fig. 1(b)]. 5, 10, and 15% (w/v) GelMA were tested in this fabrication process. 5% (w/v) GelMA was found to be too thin to maintain the shape of micropatterns, while 10 and 15% (w/v) GelMA showed acceptable formability (Fig. S1, supplementary material). Taking other material properties into consideration, 10% (w/v) GelMA was used for evaluating shape fidelity.

Surface profiles of micropatterns on silicon wafer, PDMS mold, and GelMA were visualized using a 3D measuring laser microscope [Fig. 1(c)]. Results show that micropatterns were replicated from silicon wafers to PDMS molds with high fidelity, with depth ranging from 1.5 to 15 *μ*m (Fig. S2, supplementary material). Figure 1(d) demonstrates that the depth of the micropatterns (≤10 *μ*m) decreased by approximately 50% when being transferred from PDMS to GelMA. Some minor defects were also observed. However, when the depth of the micropattern was increased to 15 *μ*m, GelMA was not able to maintain acceptable geometrical quality. This is most likely due to its viscous property and the short cross-linking time. With the increase in depth-width ratio, it becomes more difficult for GelMA to infiltrate into the corner of micropatterns in PDMS molds. Nevertheless, deep grooves might not be suitable for CMs to connect and communicate with their neighbors. Therefore, 10-*μ*m-deep micropatterns were fabricated across all the following experiments, providing sufficient guiding and enabling intercellular communication at the same time.

### B. Characterization of GelMA hydrogel

**FIG. 2.**
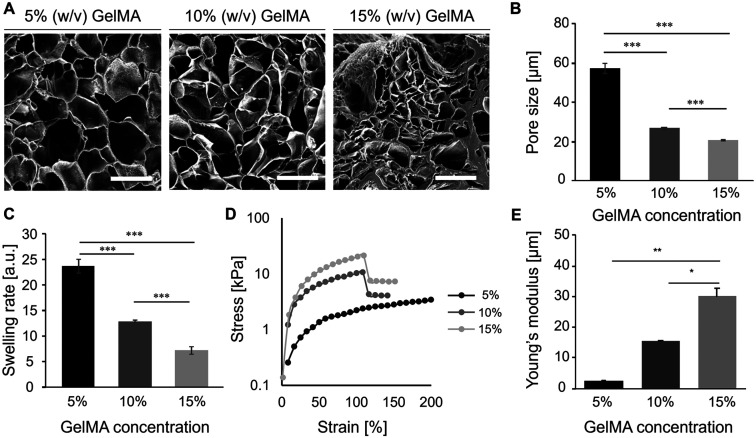
Characterization of GelMA hydrogel. (a) Representative porous microstructure of 5, 10, and 15% (w/v) GelMA by s.e.m (scale bar = 200 *μ*m). (b) Pore size, (c) swelling ratio after swelling in PBS for 24 h, (d) representative stress–strain curve, and (e) Young's modulus. Data are presented as mean ± s.e.m. (standard error of mean), n ≥ 3. Significance level denotation: ^*^p < 0.05, ^**^p < 0.01, and ^***^p < 0.001.

Microstructure, swelling performance, and mechanical property of 5, 10, and 15% (w/v) GelMA were investigated. Scanning electron microscope (SEM) images suggest that cross-linked GelMA hydrogel with higher concentration had a smaller pore size [Figs. 2(a) and 2(b)]. The smallest swelling ratio was observed in 15% (w/v) GelMA through the 120-h swelling in PBS [Figs. 2(c) and S4, supplementary material). The Young's modulus of 15% (w/v) GelMA (30.1 ± 2.5 kPa, mean ± standard error of mean) was significantly higher than that of 5% (w/v) GelMA (2.5 ± 0.4 kPa) and 10% (w/v) GelMA (15.3 ± 0.4 kPa) [Figs. 2(d) and 2(e), p < 0.05]. These results are consistent with that in previous literatures and of the same order of magnitude as that of porcine or ovine cardiac muscle tissues (47–87 kPa).^11,38^ Both 10 and 15% (w/v) GelMA show good shape fidelity in x–y plane (Fig. S1, supplementary material). Although 10% (w/v) GelMA has lower Young's modulus than 15% (w/v) GelMA, it has lower viscosity at the same time (Fig. S3, supplementary material). This results in a wider process window in terms of cross-linking time, environment temperature, etc. In addition, larger pores in 10% (w/v) GelMA are beneficial to the nutrition delivery either *in vitro* or *in vivo*. Therefore, 10% (w/v) GelMA was selected for constructing micropatterned cell-guiding substrates in the following experiments.

**FIG. 3.**
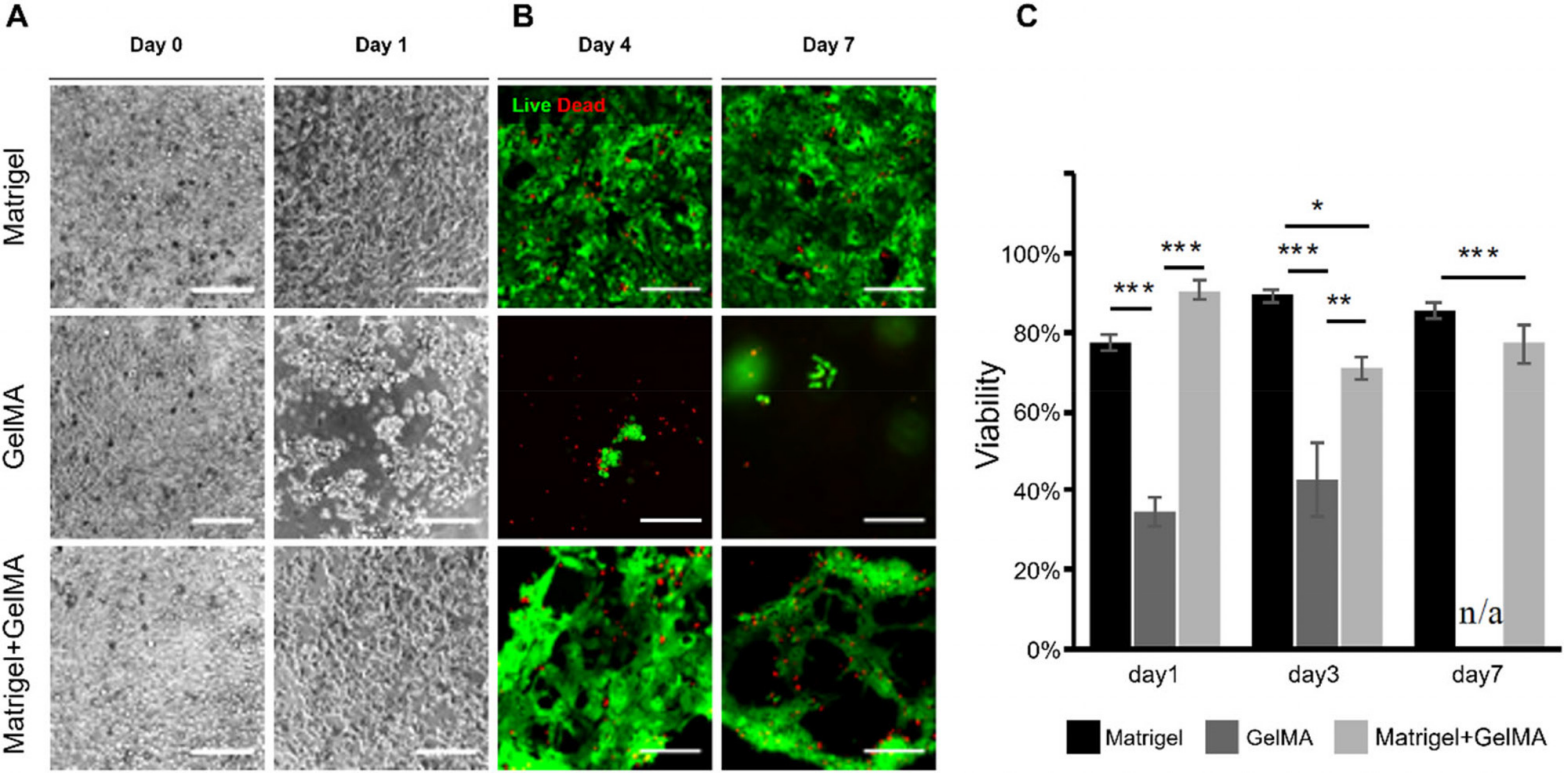
Morphology and viability of iPSC-CMs. (a) iPSC-CMs adhere, spread, and beat at day 1 after seeding on Matrigel (coated on Petri dish) and Matrigel+GelMA (Matrigel-coated GelMA), while were washed away on pristine GelMA. (b) iPSC-CMs maintained high viability until cultured for 7 days on Matrigel and Matrigel+GelMA. (c) Viability of iPSCCMs at day 1, day 4, and day 7. Scale bar = 200 *μ*m. Data are presented as mean ± s.e.m. (standard error of mean), n ≥ 3 for all other groups. Significance level denotation: ^*^p < 0.05, ^**^p < 0.01, and ^***^p < 0.001.

### C. Morphology and viability of iPSC-CMs

Matrigel, GelMA, and Matrigel + GelMA (Matrigel-coated GelMA) were compared for seeding and culturing induced pluripotent stem cell-derived cardiomyocytes (iPSC-CMs). CMs attached, spread, and started beating after being seeded on substrates for 24 h in all three conditions [Fig. 3(a)]. However, it was observed that the cell adhesion was worse on pristine GelMA than on Matrigel or Matrigel + GelMA. iPSC-CMs contracted spontaneously as a whole during the 7-day culturing on Matrigel and Matrigel+GelMA but were mostly washed away on GelMA. Live/dead assay reveals that high viability was maintained from day 1 to day 7 on Matrigel (77.6 ± 2.2% to 85.3 ± 2.2%) and Matrigel + GelMA (90.6% ± 2.6% to 77.1% ± 4.9%) [Figs. 3(b) and 3(c)]. However, the viability on GelMA substrates remained consistently low (34.5% ± 3.6% at day 1 and 46.0% ± 8.5% at day 4), and at day 7, the cell count was too low to calculate. These results show that Matrigel+GelMA is suitable as a cell culturing substrate material, while pristine GelMA might lack cytocompatibility.

**FIG. 4.**
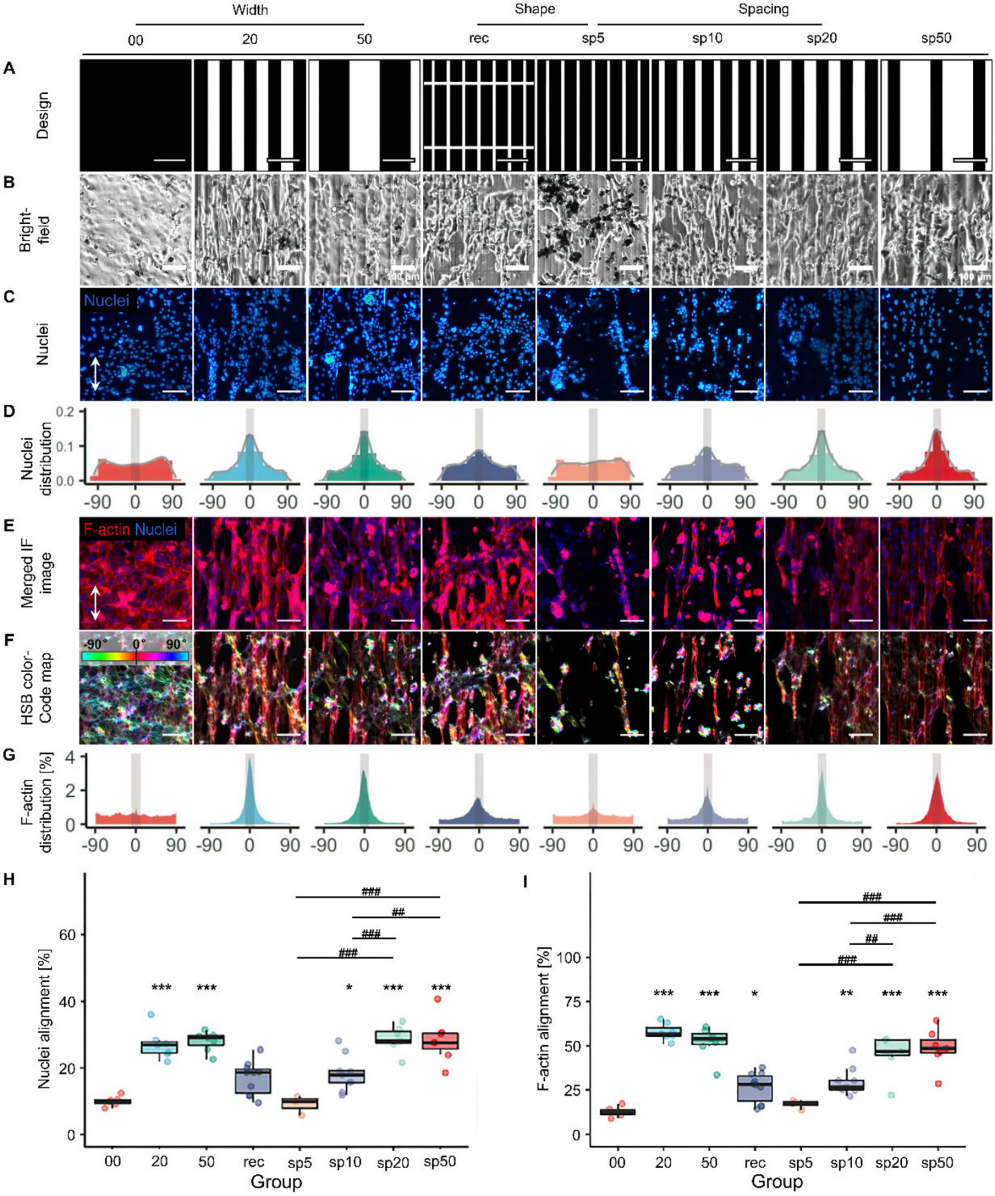
Effects of micropatterns on iPSC-CMs organization. (a) Designed micropatterns. (b) iPSC-CMs align under the guidance of seven types of micropatterns, 00 as control group. (c) Nuclei are stained with DAPI. (d) Representative histograms of the distribution of nuclei's direction. (e) Merged immunofluorescence images of F-actin and nuclei (stained with TRITC phalloidin and DAPI, respectively). (f) HSB (hue-saturation-brightness) color-coded map in which the orientation of F-actin is mapped to hue value. The color bar in the inset indicates the mapping relationship between direction and color. (g) Representative histogram of the F-actin distribution. Scale bar = 100 *μ*m in (a), (b), (c), (e), and (f). Gray ribbons in (d) and (g) show the range of 0°±10°, where the expected direction is indicated by white arrows. Cells which align in this range are defined as “aligned”. Percentage of aligned cells evaluated by (h) nuclei and (i) F-actin, respectively. Data are presented as mean ± s.e.m. (standard error of mean), n ≥ 3. Significance level denotation: ^*^p < 0.05, ^**^p < 0.01, and ^***^p < 0.001 for the comparison between each micropattern and control. ^#^p < 0.05, ^##^p < 0.01, and ^###^p < 0.001 for the comparison between micropatterns.

### D. Effects of micropatterns on iPSC-CMs organization

Seven types of micropatterns were designed to explore the influence on iPSC-CMs of three geometrical parameters, i.e., width, spacing, and shape [Fig. 4(a)]. The width parameter was investigated using group 20 and 50, denoting micropatterns that consist of 20-*μ*m-wide parallel grooves with 20-*μ*m spacing and 50-*μ*m-wide grooves with 50-*μ*m spacing, respectively. By keeping the width constant at 20 *μ*m, the spacing was set to 5, 10, 20, and 50 *μ*m, which are denoted as sp5, sp10, sp20, and sp50, respectively. The shape parameter was studied by adding interconnecting ridges between grooves in sp5, resulting in 100 × 20 *μ*m^2^ rectangular grids. This micropattern is denoted as rec. Flat substrates (00) served as the control group. The effects of these micropatterns on guiding the organization of iPSC-CMs were investigated.

**FIG. 5.**
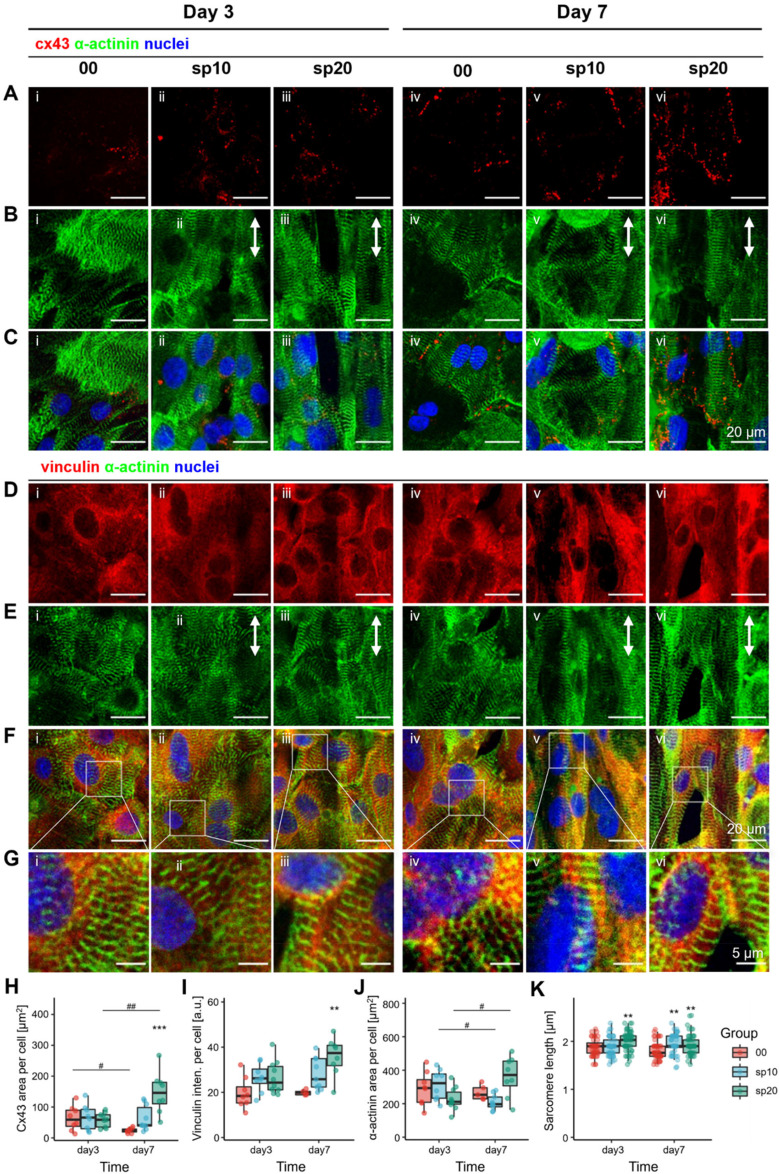
Effect of micropatterns on iPSC-CMs maturation. Immunofluorescence staining images of (a) cx43, (b, e) α-actinin, and (d) vinculin, respectively. (c) and (f) Images of merged RGB channels. (g) Magnified images of selected regions of interest in (f), showing details of sarcomeric structures. (h), (i), and (j) Cx43 area, vinculin mean intensity, and α-actinin area, respectively, that are normalized by the number of nuclei. (k) Sarcomere length. Scale bar = 20μm in (a)–(f) and 5 *μ*m in (g). In (a)–(g), (i) and (iv) denote the control group 00, (ii) and (v) denote the experiment group sp10, and (iii) and (vi) denote the experiment group sp20; (i)–(iii) show results at day 3 and (iv)–(vi) show results at day 7. White arrows in (b) and (e) indicate the direction of microgrooves. Data are presented as mean ± s.e.m. (standard error of mean), n ≥ 3. Significance level denotation: ^*^p < 0.05, ^**^p < 0.01, and ^***^p < 0.001 for comparisons within a group (00 as reference); and ^#^p < 0.05, ^##^p < 0.01, and ^###^p < 0.001 for comparisons between groups.

iPSC-CMs were cultured on these micropatterned substrates for 7 days. Majority of cells were spread and elongated in all conditions, as shown in brightfield micrographs [Figs. 4(b) and S6, supplementary material]. Nuclei demonstrated oval shape and their orientation was identified by measuring the angle between the major elliptical axis and the expected direction [Fig. 4(c)]. The orientational distribution of nuclei is shown in histograms [Fig. 4(d)] with bin width of 20°. Cytoskeletal F-actin was also stained for visualizing the conformation of iPSC-CMs [Fig. 4(e)]. Figure 4(f) shows HSB (hue, saturation, brightness)-coded micrographs where the orientation of F-actin fibers is mapped from angle to hue value. iPSC-CMs with nuclei or F-actin aligned within 0°±10° from the expected direction are defined as aligned and indicated by gray ribbons in Figs. 4(d) and 4(g). Figures 4(h) and 4(i) show the statistics of nuclei and F-actin alignment, respectively.

It is evident that cells on the hydrogel surface with microgrooves exhibit a pronounced concentrated distribution at 0° ± 10° [Fig. 4(d)]. As shown in the quantitative analysis results [Fig. 4(h) and Table I], The group 20 and 50 have 27.0% ± 1.7% and 28.0% ± 1.1% of cells oriented. In comparison, the control group has only 10% ± 0.6%. Statistical analysis reveals a significant difference between the group 20 and 50 compared to the control group (p < 0.001), while there was no difference between the two experimental groups. It can be concluded that microgrooves significantly affect cell alignment, with no significant difference in the inducing effect between 20 and 50 *μ*m groove widths. The probability distribution of F-actin alignment direction is consistent with the nuclear orientation [Fig. 4(g)]. The statistical analysis results [Fig. 4(i)] also confirm the significant impact of microgrooves on cell cytoskeletal protein alignment. From the probability distribution, the arrangement of cytoskeletal proteins in the 20 *μ*m groove group is more concentrated, with a higher peak.

**TABLE I.** Aligned proportion of iPSC-CMs on various micropatterns.

Group	00	20	50	rec	sp5	sp10	sp20	sp50
Aligned proportion of nuclei (%)	10.0	27.0	28.0	17.0	9.1	18.3	28.7	28.4
(mean ± s.e.m.)	±0.6	±1.7	±1.1	±1.6	±1.7	±1.8	±1.5	±2.6
Aligned proportion of F-actin (%)	12.8	58.0	52.1	26.7	17.2	30.0	45.7	48.7
(mean ± s.e.m.)	±1.1	±1.8	±3.4	±2.7	±1.2	±2.6	±4.2	±4.2

Selecting 20 *μ*m-width groove as a controlled variable, the spacing was changed to 5, 10, 20, and 50 *μ*m to investigate the influence of spacing on the induction efficiency [Fig. 4(d)]. The highest proportion of aligned CMs (28.7% ± 1.5%) is observed in 20-μm spacing (sp20). Increasing or decreasing the spacing both resulted in lower organization proportion. This proportion is 28.4% ± 2.6% in sp50 (not significant) and 17.0% ± 1.6% in sp10 (p < 0.001).

The highest proportion of aligned cardiac muscle cells (28.7% ± 1.5%) was observed at a 20 *μ*m spacing (sp20). Increasing or decreasing the spacing resulted in lower tissue proportions. The proportion was 28.4% ± 2.6% (not significant, p < 0.001) in sp50 and was 17.0% ± 1.6% in sp10. Compared to the control group, the proportions of cell alignment in the sp10, sp20, and sp50 groups were all significantly higher (p < 0.05). Interestingly, sp5 micropattern worsened the alignment of CMs when compared with the control group, with no statistical significance. Furthermore, the induction efficiency did not exhibit a significant difference between adjacent groups (sp5 and sp10, sp10 and sp20, and sp20 and sp50). However, there were significant differences between non-adjacent groups (p < 0.05). The influence of groove spacing on cell alignment was further confirmed by quantitative analysis of F-actin.

Based on 20 *μ*m-wide grooves with 5 *μ*m spacing, rec added 5 *μ*m wide ridges every 100 *μ*m to examine the effect of shape factors (microgrid vs microgroove) on inducing cell alignment. Statistical analysis results indicate that there was no significant difference in the induction efficiency between rec and sp5, and these two groups also did not differ from the control group [Fig. 4(h)]. In contrast to the nuclear orientation, the proportion of F-actin alignment in rec was significantly higher than in the control group (p < 0.005). This suggests that the ridges oriented perpendicular to the microgroove direction induce a higher degree of alignment of cell cytoskeletal proteins. The possible reason is that these ridges provide more adhesion sites for the cells, making them more inclined to align in the induced direction during the contraction–relaxation process.

### E. Effects of micropatterns on iPSC-CMs maturation

Two types of micropattern were chosen for investigating their effects on iPSC-CMs' maturation. One was sp20, which led to the highest alignment proportion in all aforementioned micropatterns. The other was sp10 that had moderate performance. Flat substrate (denoted as 00) was used as control. Immunofluorescence staining was carried out for connexin 43 (cx43), vinculin, and sarcomeric α-actinin at day 3 and day 7, respectively [Figs. 5(a)–5(g)]. Quantitative analysis was sequentially conducted for the average intensity of vinculin, the average area of cx43 and α-actinin.

**FIG. 6.**
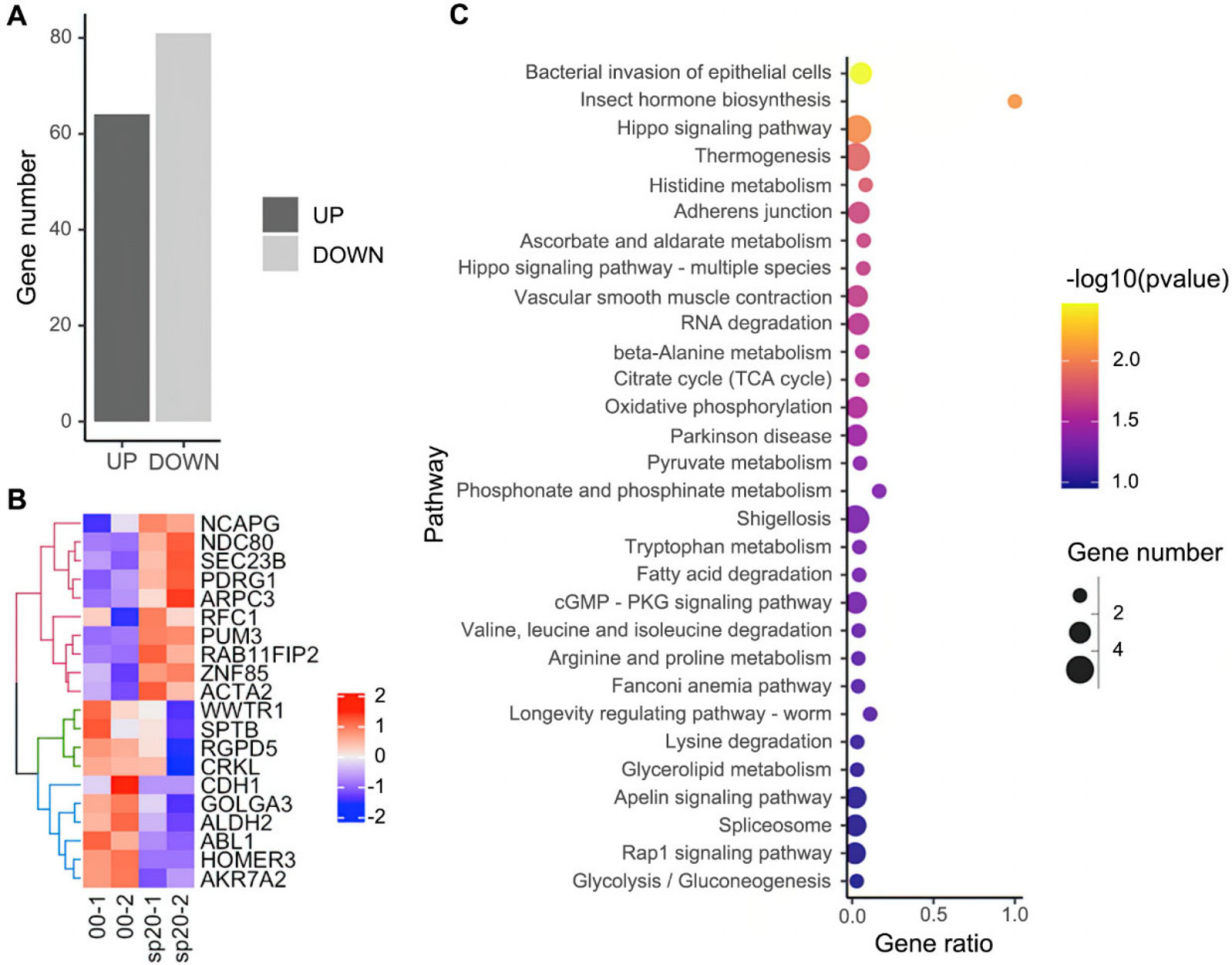
RNA-sequencing results. (a) The number of up- and down-regulated genes. (b) Heatmap of top 20 significantly differently expressed genes. (c) Kyoto Encyclopedia of Genes and Genomes (KEGG) analysis of enriched signaling pathways.

Myofibril maturation is a hallmark of CMs maturation. Sarcomeres are subunits of myofibrils and are longitudinally repeated in myofibrils.^40,41^ We used stained α-actinin to reflect sarcomeric alignment and expansion. In the control group, the amount of α-actinin expression [Fig. 5(j)] did not show significant differences among groups. In the sp20 group, however, there was a significant increase in expression with the extension of culture time (p < 0.05). Notably, the expression in the sp10 group showed a significant decrease (p < 0.05). This result may be attributed to the differential influence of micropatterns on maturation of CMs. In the sp10 group, cells might align more compactly, reducing the α-actinin area per cell. Conversely, cells might align more loosely, leading to a larger α-actinin area per cell in the sp20 group. The average sarcomere length [Fig. 5(k)] of iPSC-CMs in sp20 is significantly larger than that in the control group at both day 3 (2.01 ± 0.02 vs 1.86 ± 0.03 *μ*m, p < 0.01) and day 7 (1.93 ± 0.03 vs 1.81 ± 0.03 *μ*m, p < 0.01). As a comparison, the average sarcomere length in sp10 is significantly larger than that in the control group only at day 7 (1.95 ± 0.03 vs 1.81 ± 0.03 *μ*m, p < 0.01). This result suggests that the better organized iPSC-CMs have increased sarcomere length.

It is noteworthy that the differences in α-actinin area per cell at days 3 and 7 between the sp10 and sp20 groups showed an opposite trend. Specifically, despite having a similar sarcomere length to sp10, sp20 exhibited an increase in α-actinin area per cell at day 7 compared to day 3. One possible explanation for this observation could be related to the maturation state of the CMs. While α-actinin is a marker of sarcomeric organization, the area of α-actinin may not be directly correlated with sarcomere length at all stages of cell maturation. The increase in α-actinin area at day 7 may indicate a more advanced state of sarcomeric organization and maturation in sp20, where the sarcomeres are more tightly packed and organized, increasing the total α-actinin area required. This contrasts with sp10, where a smaller α-actinin area per cell may indicate a different maturation trajectory.

Aligned iPSC-CMs have higher level of maturational integration into tissues due to the contact guidance of micropatterns. Meanwhile, sp20 outperforms sp10 in this regard. Maturational integration of CMs into tissues require the formation of intercalated disks (ICDs) and the attachment to ECM through costameres.^40^ Cx43 is a major component of gap junctions, which compose ICDs and mediate intercellular communication. In addition, ICDs and costameres also contribute to CMs' maturation by harboring vinculin-based actomyosin organizers. These organizers play important roles in sarcomere assembly and potentially in mediating sarcomere expansion.^42^

Our results show that the expression of cx43 and vinculin has no significant difference at day 3 among three groups [Figs. 5(h) and 5(i)]. However, at day 7, the amount of cx43 expression and vinculin expression in sp20 are significantly higher than that in the control group (147.1 ± 23.9 vs 24.1 ± 2.8 *μ*m^2^ and 35.8 ± 3.0 vs 19.9 ± 0.5, p < 0.01). This change suggests that aligned iPSC-CMs show improved formation of ICDs and costameres, implying higher level of maturational integration to cardiac tissues. In addition, temporal comparisons show a decrease in cx43 expression in the control group (p < 0.05) and an increase in sp20 (p < 0.01) from day 3 to day 7, but no difference in sp10. No significant difference is observed in vinculin expression. This implies that contact guidance of micropatterns has more profound influence on intercellular integration than cell-ECM attachment.

RNA sequencing was employed to further investigate the effect of micropatterns. Among 20286 identified genes, 64 genes were up-regulated and 81 were down-regulated in sp20, compared with the control [Fig. 6(a)]. Top 20 significantly differently expressed genes are shown in the heatmap [Fig. 6(b)]. Kyoto Encyclopedia of Genes and Genomes (KEGG) analysis revealed 30 significantly enriched signaling pathways [Fig. 6(c)]. It can be observed that there are significant differences in the pathways related to the formation of adherens junctions, which are associated with intercellular gap formation. Although not directly stained, their importance in the formation of cell gaps is related to the physical alignment and structure of cells observed in immunofluorescence staining. Changes in adherens junctions may affect the spatial arrangement of CMs, thus coordinating with the tissue structural changes revealed by immunofluorescence staining. Additionally, significant differences were observed in signaling and metabolic pathways. Signaling pathways are closely associated with functions such as stress response, metabolic regulation, and growth factor signaling in CMs. Metabolic pathways, particularly glycolysis, the citrate cycle, and oxidative phosphorylation, are the primary routes for cells to generate energy (ATP). The maturation and functional maintenance of CMs depend on the efficient operation of these pathways. Thus, the KEGG analysis to some extent validates the positive role of micropatterns in inducing the organizational and functional maturation of CMs, corroborating the conclusions of the immunofluorescence staining analysis.

**FIG. 7.**
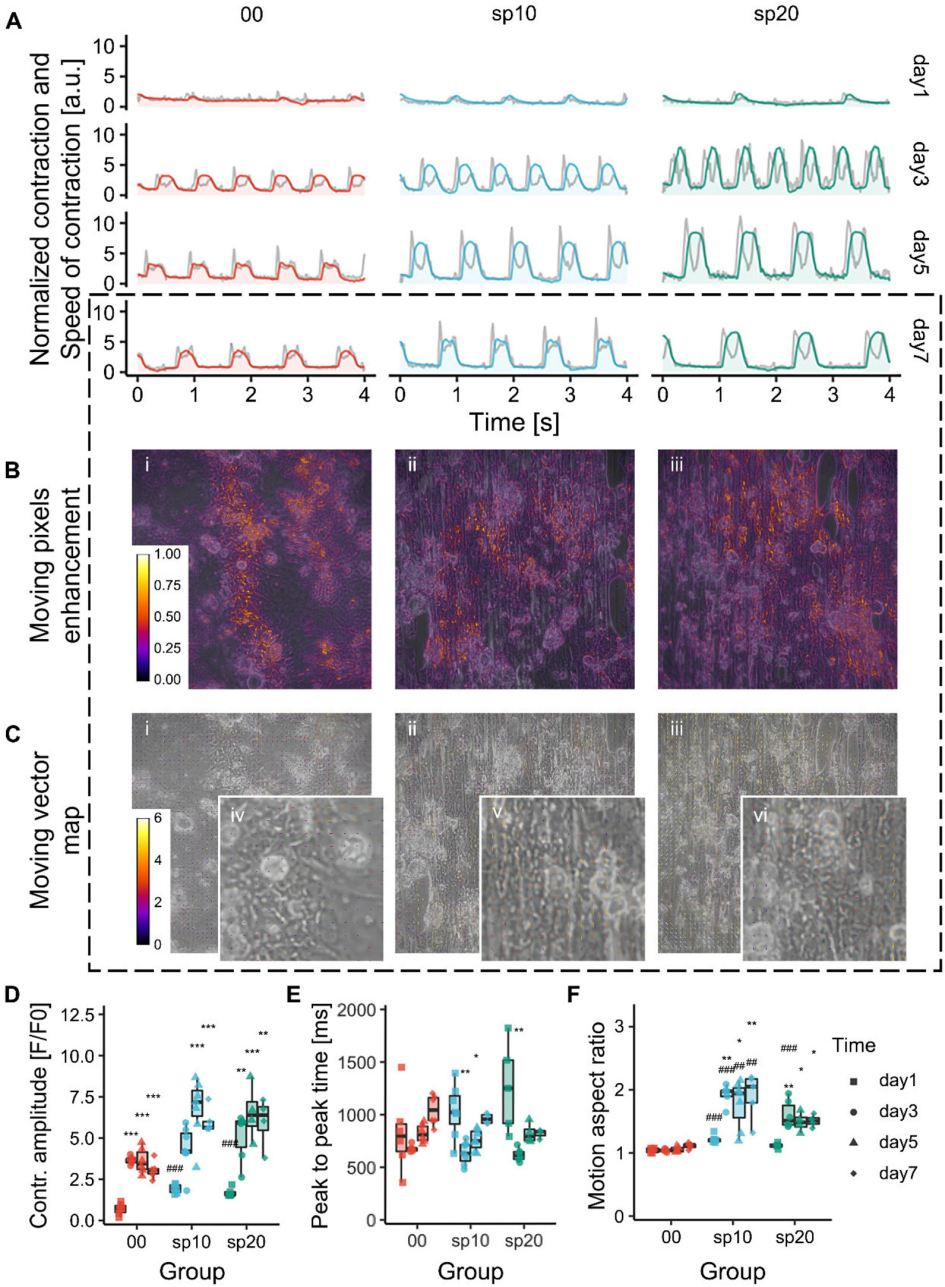
Effect of micropatterns on iPSC-CMs contraction. (a) Representative beating profiles of iPSC-CMs at day 1, 3, 5, and 7, respectively. Values are normalized by the mean of day 1 data in each group. Gray lines demonstrate the speed of contraction. (b) The difference in gray value between the selected baseline frame and peak frame at each pixel is enhanced by “Fire” LUT in the ImageJ software, showing the relative intensity of beating. (c) Motion vector map are generated using block-matching algorithm, demonstrating the direction and displacement [unit: pixel] of beating motion. (i) and (iv) in (b) and (c) denote the control group, (ii) and (v) sp10, and (iii) and (vi) sp20. (d) Contraction amplitude that is normalized by the baseline value in each condition. (e) Peak to peak time. (f) Motion aspect ratio that is defined as the average ratio between the displacement in y and x direction. Ratio = 1 denotes isotropic motion. Data are presented as mean ± s.e.m. (standard error of mean), n ≥ 3. Significance level denotation: ^*^p < 0.05, ^**^p < 0.01, and ^***^p < 0.001 for comparisons within a group (day 1 as reference); and ^#^p < 0.05, ^##^p < 0.01,and ^###^p < 0.001 for comparisons between groups (00 as reference).

### F. Effects of micropatterns on iPSC-CMs contraction

To investigate the contractile behavior of iPSC-CMs under the guidance of micropatterns, beating CMs were filmed. CardioMotion and MotionVector plugins were developed in the NIH ImageJ software to analyze the contractile motion from these movies (Fig. 7; Movies S1 and S12, supplementary material).

**FIG. 8.**
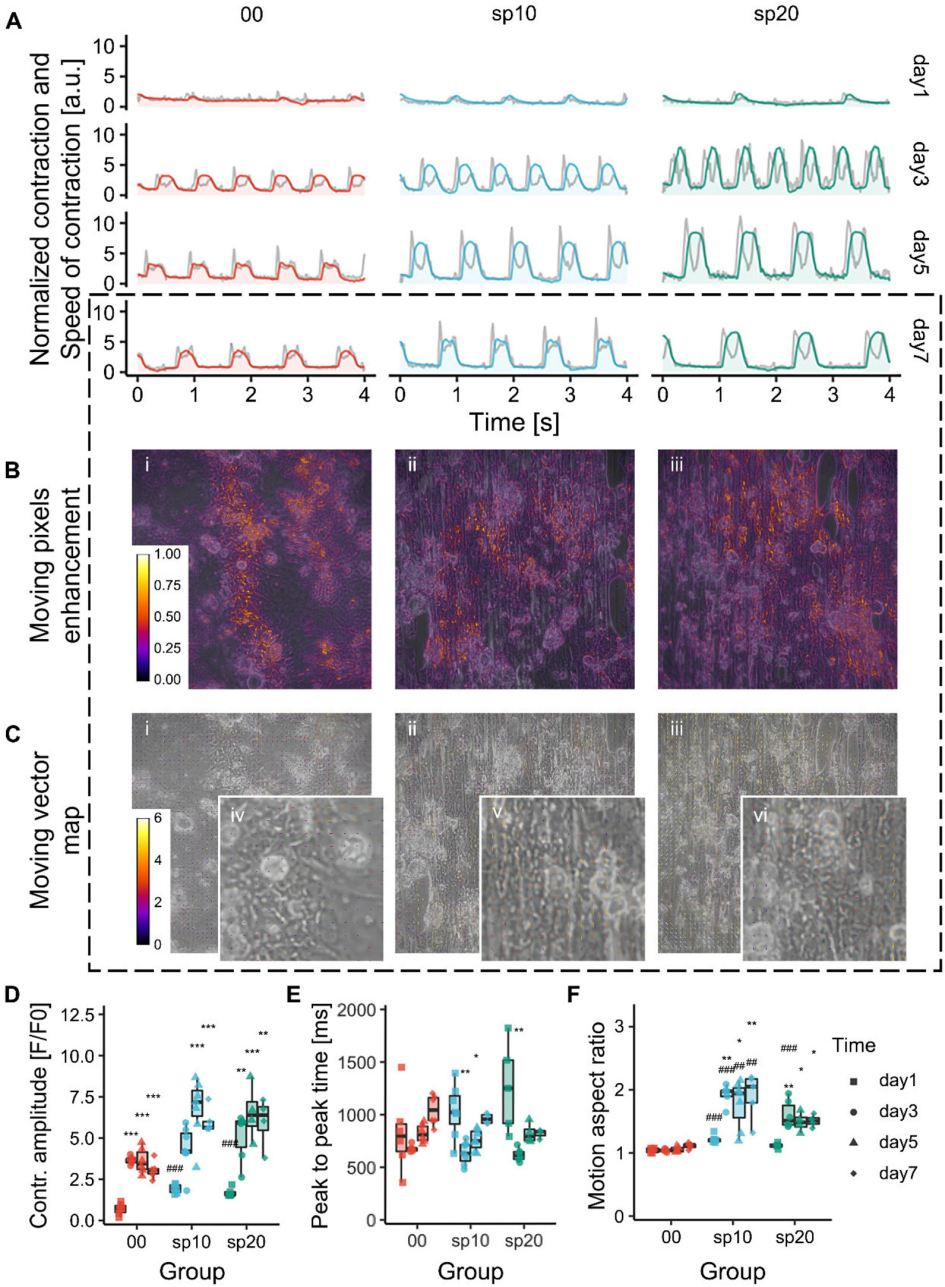
Effect of micropatterns on iPSC-CMs contraction. (a) Representative beating profiles of iPSC-CMs at day 1, 3, 5, and 7, respectively. Values are normalized by the mean of day 1 data in each group. Gray lines demonstrate the speed of contraction. (b) The difference in gray value between the selected baseline frame and peak frame at each pixel is enhanced by “Fire” LUT in the ImageJ software, showing the relative intensity of beating. (c) Motion vector map are generated using block-matching algorithm, demonstrating the direction and displacement [unit: pixel] of beating motion. (i) and (iv) in (b) and (c) denote the control group, (ii) and (v) sp10, and (iii) and (vi) sp20. (d) Contraction amplitude that is normalized by the baseline value in each condition. (e) Peak to peak time. (f) Motion aspect ratio that is defined as the average ratio between the displacement in y and x direction. Ratio = 1 denotes isotropic motion. Data are presented as mean ± s.e.m. (standard error of mean), n ≥ 3. Significance level denotation: ^*^p < 0.05, ^**^p < 0.01, and ^***^p < 0.001 for comparisons within a group (day 1 as reference); and ^#^p < 0.05, ^##^p < 0.01,and ^###^p < 0.001 for comparisons between groups (00 as reference).

Motion analysis of beating iPSC-CMs suggested a correlation between contraction performance and organization. Figure 7(a) shows representative beating profiles of iPSC-CMs from day 1 to day 7. Each profile was selected out of at least three similar replicates at each time point under each condition. The temporal profiles of contraction and speed of contraction under each condition were normalized with respect to the mean value at day 1. Contractile characteristics, including amplitude, peak-to-peak time, and motion aspect ratio, were then analyzed.

Generally, the contraction amplitude of micropattern-guided iPSC-CMs (through 7-day culturing) is significantly higher than that in the control group [Fig. 7(d)]. In addition, the amplitude of micropattern-guided CMs increased from day 1 to day 5, then decreased at day 7. Contrarily, this turning point appeared earlier at day 3 in the control group. This suggests that the contractile capability of well-organized iPSC-CMs was improved and maintained for longer time under the guidance of the micropatterns.

The guidance of micropatterns was found to have minimal effect on beating rate, that is the reciprocal of peak-to-peak time [Fig. 7(e)]. Micropattern-guided iPSC-CMs in sp10 and sp20 did not show significantly different beating rate from those in the control group. The peak-to-peak time in all three groups exhibited the same major trend during the experiment, i.e., decreasing from day 1 to day 3, then increasing until day 7. The only exception is found at day 7 in sp20 (816.3 ± 27.0 ms), which was negligibly smaller than that at day 5 (820.6 ± 48.1 ms). However, most of in-group comparisons exhibited no significant differences. The increase in peak-to-peak time from day 3 to day 7 suggested enhanced maturation of iPSCCMs.^43^ We speculate that the abnormally large peak-to-peak time at day 1 resulted from the deficiency of the CardioMotion tool. iPSC-CMs might not completely recover after being seeded on the GelMA substrates for only 1 day. Therefore, their contraction was weak at day 1 when compared to noise, the tool may be not able to accurately extract the actual values. This explanation is also supported by the large variance of the data at day 1.

The MotionVector software was developed for analyzing the motion anisotropy of contracting iPSC-CMs. Briefly, this software uses a block-matching algorithm to generate a map of motion vectors of filmed beating CMs and calculate the overall motion aspect ratio (Fig. S9, supplementary material).^44^ Figs. 7(b) and S10 (supplementary material) show static frames of beating CMs with moving pixels highlighted. Larger highlighted area in sp10 and sp20 suggests that well-organized CMs exhibited larger contractile displacement. Motion vector maps are shown in Figs. 7(c) and S11 (supplementary material). Well-organized CMs in sp10 and sp20 exhibited significantly higher motion anisotropy than randomly distributed ones in the control group. This is proved by the statistical analysis of motion aspect ratio, as shown in Fig. 7(f). In addition, motion aspect ratio in sp10 and sp20 increased along culturing, while those in the control did not.

## III. DISCUSSION

This study elucidates the multi-parameter effects of width, spacing, and shape on the contact guidance effect of micro-patterns on cells. This effect manifests in three aspects: cell orientation, functional maturation, and synchronous beating. By quantitatively analyzing the induction efficiency of seven types of micro-patterned hydrogel patches on cell orientation, it was confirmed that microgrooves with widths and spacings both set at 20 *μ*m exhibit the highest induction efficiency, which has also been validated in other studies.^45^ Width and spacing have a significant impact on the induction ability of micro-patterns, while the influence of shape is limited. CMs oriented by micro-patterns not only demonstrate a higher degree of maturity at the cellular level but also experience enhanced integration into mature cardiac tissues. Specifically, compared to the control group (randomly oriented CMs), CMs induced by micro-patterns show a 6.7% increase in sarcomere length, a 510% and 80% increase in the average expression levels of cx43 and vinculin, respectively. The amplitude of CMs beating is significantly enhanced, and the consistency of beating direction is also significantly improved.

In addition to micro-molding, 3D printing has also been employed for the fabrication of micropatterns. First, this technology allows for convenient modification of the structure and geometry of patterns, enabling efficient study of the impact of different structures on cell alignment. Second, it provides the possibility of constructing complex three-dimensional structures using different cells and materials. Tijore *et al.* utilized extrusion-based 3D printing to print gelatin filaments with a diameter of approximately 300 μm on gelatin film surfaces, using the grooves formed between the filaments to constrain cell alignment.^14^ The results showed that grooves with actual widths of around 200–300 *μ*m had a significant inducing effect on cell alignment direction. Lind *et al.* utilized extrusion-based 3D printing to prepare surfaces with periodic undulations, with periods ranging from 40 to 100 *μ*m. Among these, a period of 60 *μ*m resulted in a larger proportion of cells aligning directionally.^46^ Overall, the resolution of extrusion-based 3D printing is relatively low compared to micro-molding, and micropatterns manufactured using hydrogel materials as printing substrates have dimensions larger than the width of CMs (approximately 20 *μ*m), thus resulting in relatively limited inducing effects.

CMs showed higher degree of organization on micropatterned substrates compared to unpatterned control. The phenomenon of contact guidance effect was confirmed by the significantly higher alignment proportion in group 20 and 50 than that in the control group (p < 0.001). However, there is no significant difference between the proportion of aligned nuclei in group 20 and 50. This contradicts to what was previously reported, where 20 × 20 *μ*m micropattern significantly outperformed 50 × 50 *μ*m.^27^ Although the depth and the surface profile of micropatterns were not reported in Ref. 27, we suspect that these factors may lead to the difference. Fu *et al.* reported that 20-*μ*m microgrooves had more significant guiding effect on neonatal rat CMs than 40-μm or 60-μm ones, while they set the spacing between grooves constant at 30 μm.^45^

It should be noted that there are significant differences between different types of cells.^47^ As we reviewed previously,^25^ most studies on contact guidance of CMs using micropatterns use neonatal rat CMs rather than iPSC-CMs.^14,27,28,45^ Therefore, while tissue microstructures resembling those of the native rat heart have been reported,^13^ iPSC-CMs hold advantages in clinical applications and personalized medicine due to their human cardiac electrophysiological characteristics and the biochemical and molecular biology properties of human cardiac muscle cells.^48^

In addition, the criteria for evaluating the degree of cell alignment are quite different from one literature to another. For example, most studies use the proportion of aligned cells as the criteria, while some use orientational order parameter (OOP).^46^ Furthermore, the standard for defining “aligned cell” varies from 0°±5° to 0°±30° in different studies. These factors result in difficulty in comparing the guiding effect of different micropatterns.

There are several limitations of this study. First, the micropatterns used in our study were not thoroughly characterized in terms of their depth and profile. Although we successfully created micropatterns with different groove widths and spacings, we did not investigate variations in their depth or profile. It is plausible that these aspects could have a significant impact on the alignment and maturation of CMs. Future studies should strive to provide a comprehensive characterization of the micropatterns employed. Second, while we observed positive effects on the alignment and functional maturation of iPSC-CMs, we did not conduct a comprehensive analysis of factors associated with maturation, such as intercalated disk formation, calcium handling, or sarcomere organization. For a more comprehensive understanding of how micropatterned substrates influence CMs maturation, future studies should delve into these aspects in greater details. Additionally, although we demonstrated positive effects of micropatterns on CMs alignment and maturation over a 7-day period, the long-term effects were not explored. Investigating the stability and persistence of these effects over more extended culture durations is crucial to assess the long-term utility of micropatterns in cardiac tissue engineering.

## IV. CONCLUSION

Our study investigated the impact of various micropatterned GelMA substrates on the organization, maturation, and contraction of iPSC-CMs through contact guidance. Our findings indicate that by manipulating the geometric parameters of these micropatterns, including width, spacing, and shape, we can effectively influence the alignment of iPSC-CMs. It has been revealed that among the micropattern designs explored, the 20-*μ*m-width and 20-*μ*m-spacing microgrooves emerged as the most promising. Several lines of evidence support this conclusion. First, this specific micropattern design yields the highest degree of cell alignment, as demonstrated by our analysis of nuclei and F-actin orientation. Second, iPSC-CMs cultured on these micropatterned GelMA substrates exhibit signs of improved functional maturation, including increased expression of connexin43 and vinculin, as well as extended sarcomere length. Third, the micropatterned GelMA substrates enhance the contractility of iPSC-CMs, manifesting as increased contractile amplitude and anisotropy, further emphasizing their similarity to highly aligned iPSC-CMs and native cardiac tissues.

Importantly, our study represents the first systematic exploration of various geometric parameters and their effects on regulating the functions of iPSC-CMs. By applying consistent criteria to assess the organization, maturation, and contraction of iPSC-CMs, our findings offer valuable insights into optimizing cell-guiding micropatterns and advancing the development of functionally mature cardiac tissues *in vitro*. These results hold significant promise for the fields of cardiac tissue engineering and regenerative medicine, providing valuable perspectives for future research in areas such as cell therapy and drug screening. Nonetheless, it is essential to underscore the need for further research to deepen our understanding of how different geometric parameters impact the functionality of iPSC-CMs and their potential applications in clinical settings.

## V. METHODS

### A. GelMA hydrogel preparation

Gelatin methacrylate (GelMA, lyophilized, ∼60% methacryloyl substitution) and blue light source (3 W, 405 nm) were purchased from Suzhou Intelligent Manufacturing Research Institute (Suzhou, China). The photoinitiator (lithium phenyl-2,4,6 trimethylbenzoylphosphinate, LAP) was purchased from Tokyo Chemical Industry Co., LTD. (Tokyo, Japan). LAP solution was prepared by dissolving LAP powder in phosphate-buffered saline (PBS, Hangzhou Genom Biomedical Technology Co., Ltd., Hangzhou, China) and filter sterilized by Millex-GP syringe filter unit with a 0.22 *μ*m pore size (Merck Millipore, Massachusetts, United States). The lyophilized GelMA was sterilized by UV for at least 30 min before being dissolved in the sterilized LAP solution. GelMA-LAP mixtures were stirred at 37 °C for obtaining uniform solution. The final concentrations of GelMA hydrogels were 5, 10, and 15% (w/v), respectively. The concentration of LAP was kept constant at 0.5% (w/v).

Matrigel (Corning No. 354277) was diluted to 100× with PBS and added to the surface of the GelMA hydrogel to submerge the GelMA matrix. The matrix is then incubated at 37 °C overnight. Afterward, the excess solution is aspirated, and the hydrogel is washed with culture medium to remove any unbound substances.

### B. Micropattern fabrication and characterization

The process of fabricating micropatterned GelMA hydrogel substrates is shown in Fig. 1(a). Micropatterns were designed and fabricated on silicon wafers via lithography (Mask Aligner MA6-BSA, Karl Süss, Garching, Germany) and ion etching (Oxford Plasmalab System100, GBP3260, Abingdon, United Kingdom). Polydimethylsiloxane (PDMS, SYLGARD™ 184 Silicone Elastomer Kit, Dow Chemical Company, Michigan, USA) was prepared at a 10 (base) to 1 (curing agent) mix ratio. PDMS mold was fabricated by casting the PDMS mixture over the silicon wafer and curing at 65 °C for 11–16 h. Then, uncrosslinked GelMA hydrogel (kept at 37 °C) was drop-dispensed on an unprocessed silicon wafer (kept at 20 °C) and covered with the PDMS mold. Cover glasses (thickness of 180 *μ*m) were placed aside the GelMA drop to keep the thickness of GelMA substrates consistent. After cooling at 20 °C for 30 s, GelMA was photocrosslinked for 30 s by the light source (3 W, 405 nm) that was placed 10 mm directly above the PDMS mold. Micropatterned GelMA substrates were then peeled off the mold. The surface profile of micropatterned GelMA substrate was measured using a 3D measuring laser microscope (LEXT OLS4100, Olympus, Japan).

### C. Swelling ratio, microstructure, and pore size assessment

GelMA hydrogels were drop-dispensed onto a PDMS substrate and photocrosslinked to form drop-shaped samples (200 *μ*l for each sample, diameter = 10 mm, n ≥ 3). These samples were then immersed in PBS at 37 °C for 4, 8, 24, 48, and 120 h, respectively. After being removed from PBS, the residual liquid was gently removed, and samples were weighted for obtaining the swollen weight (Ws). The dry weight (Wd) was recorded after the samples being lyophilized for at least 24 h. The swelling ratio was defined as Q = (Ws−Wd)/Wd. Samples for microstructure and pore size assessment were lyophilized for at least 48 h and viewed under a scanning electron microscope (SEM, ZEISS GeminiSEM 300, Germany). Pore sizes were determined in ImageJ software (National Institutes of Health, USA).

### D. Mechanical properties' assessment

Mechanical properties of GelMA hydrogels were assessed by uniaxial tensile tests using a dynamic mechanical analysis instrument (ElectroForce, TA Instruments, USA). Dumbbell-shaped samples were prepared and photocrosslinked using prefabricated molds. The dimensions of the narrow section were 2 × 2 × 10 mm^3^. Uniaxial tensile tests were carried out at 25 °C with a stretch rate of 1 mm·min-1. Young's moduli were calculated from the initial 2%–10% of the linear range of stress–strain curves (n ≥ 3).

### E. Cell culture and viability assessment

iPSC-CMs, culture medium, and dissociation medium were purchased from the Nanjing Help Stem Cell Innovations Co., Ltd. (NovoCell™-Cardiomyocytes Kit, HELP4111-h, Nanjing, China). iPSC-CMs were characterized by flow cytometry, immunofluorescence, and patch clamp technique before experiments (Fig. S5, supplementary material). The purity of iPSC-CMs was approximately 97%. After being recovered in the 37 °C, 5% CO_2_ incubator for at least 24 h, cells were gently digested with the dissociation medium. The dissociated cells were then centrifuged and resuspended in the culture medium. 8800 cells⋅mm^−2^ were seeded onto the surface of the pre-prepared Matrigel-coated GelMA substrates in 24-well plates. After the adherence of the cells, the culture medium was exchanged every 2 days. The viability of iPSC-CMs was assessed using Live/Dead Viability Kit (Thermo Fisher Scientific Inc., Massachusetts, United States) at day 1, day 4, and day 7, respectively. Fluorescent images were acquired using an inverted microscope (Leica DMi8, Germany).

### F. F-actin and nuclei staining

At day 7, cell-seeded GelMA substrates were rinsed with PBS, fixed, and then stained with phalloidin conjugated with TRITC (tetramethylrhodamine isothiocyanate, Shanghai Fushen Biotechnology Co. Ltd., China) and 4′,6-diamidino-2-phenylindole (DAPI, Sigma-Aldrich, Missouri, USA) to visualize F-actin and nuclei. Fluorescent images were acquired using an inverted microscope (Leica DMi8, Germany). 3–10 regions of interest from at least three independent replicates were acquired under each condition for quantitative and statistical analysis.

### G. Nuclei alignment analysis

Fluorescent images were processed using ImageJ 1.51n (National Institutes of Health, USA), following a previously reported method.^13^ Images of DAPI-stained nuclei were first converted to 8-bit grayscale images. Nuclei were selected by adjusting threshold, and clusters of nuclei were separated using “watershed” command. Each nucleus was fitted as an ellipse. The angle between the major elliptical axis and the expected direction (e.g., along the microgrooves) was measured by “analysis particles” command. Finally, the distribution of the nuclei alignment angle was calculated. Nuclei in at least three micrographs from three replications per condition were quantified. Fluorescent images were taken at 10×objective magnification so that 240–1500 nuclei were included in each micrograph.

### H. F-actin alignment analysis

The alignment of F-actin was evaluated using OrientationJ 2.0.4 (a directional image analysis ImageJ plugin), as described previously.^49^ Fluorescent micrographs of F-actin were taken at the same fields as those of nuclei. They were first converted to 8-bit grayscale images and then processed using OrientationJ with a local window of 5 pixels. The orientation of F-actin fibers was mapped to hue value in HSB (hue-saturation-brightness) color system for visualization, with red indicating 0° and cyan indicating ±90° [Fig. 4(e)].

### I. Immunofluorescence staining and imaging

For better demonstration, the cell density in this experiment was a half of the F-actin/nuclei staining, i.e., ∼4400 cells⋅mm^−2^. All staining procedures were applied using a well-established protocol. Briefly, substrates seeded with iPSC-CMs were first fixed in 4% paraformaldehyde for 30 min, permeabilized in PBS containing 0.1% (v/v) Triton X-100 for 15 min and blocked in 10% BSA for 1h. Then, immunofluorescence double-staining of α-actinin and connexin-43 (or vinculin) were performed by incubating the substrates in a mixture of mouse anti-α-actinin (1:200; Abcam, Cambridge, MA), rabbit anti-connexin-43 (1:400; Cell Signaling Technology, Danvers, MA), or rabbit anti-vinculin (1:400; Abcam, Cambridge, MA) primary antibodies overnight at 4 °C, followed by incubating with goat anti-mouse IgG conjugated with Alexa Fluor 488 (1:200; Abcam, Cambridge, MA) and goat anti-rabbit IgG conjugated with Alexa Fluor 594 (1:200; Abcam, Cambridge, MA) secondary antibodies for 1h at room temperature in the dark. After staining the nuclei with Hoechst (1:2000; Abcam, Cambridge, MA), a confocal-laser scanning microscope (TCS SP8, Leica, Germany) was employed to capture the images. For bright-field images, the exposure time was set to 38 ms with a gain of 0. For nuclei images, the exposure time was 19 ms with a gain of 100, and for merged immunofluorescence images, the exposure time was 440 ms with a gain of 100. All images were captured using the maximum aperture, with a 10× eyepiece and a 10× objective lens. 5–10 regions of interest (ROI) without overlapping from at least three independent replicates were acquired under each condition for quantitative and statistical analysis.

### J. Quantitative analysis of immunofluorescence image

Single channel images were processed using an ImageJ macro tool that was specifically developed for this study. Briefly, red (cx43 or vinculin) and green (α-actinin) channel images were first automatically thresholded using “moments” method. Then, the area and mean intensity of these thresholded regions were measured. Blue channel (nuclei) images were used for manual nuclei counting. Area of cx43 or α-actinin and mean intensity of vinculin were normalized using the number of nuclei in each ROI. The reason for using different measurements is that cx43 and α-actinin were distributed dot-wise or band-wise, respectively, while vinculin occupies the whole ROI.

### K. Sarcomere length measurement

Sarcomere length was measured using a power spectral density (PSD) method (Fig. S7, supplementary material). For each ROI, at least three linear selections were made at clearly banding areas [Fig. S7(a)]. Each linear selection crossed 3–5 sarcomeres, as Fig. S7(b) shows. Figure S7(c) exemplifies the process of extracting the dominant wavelength from a standard sinusoidal wave. Figure S7(d) shows the same process using the gray value profile that was obtained from Fig. S7(b). Briefly, gray values at linear selections were plotted with respect to its spacial position. A lowpass filter was applied to the waveform to eliminate noise. The dominant frequency *fD* was extracted using the PSD method. Sarcomere length *l* was calculated as *l* = *δ*/*fD*, where *δ* = 0.07 *μ*m is the resolution of micrographs. The microscope was set to an exposure time of 440 ms, a gain of 100, using the maximum aperture, with a 10× eyepiece and a 40× objective lens.

### L. Video acquisition and processing

Beating iPSC-CMs that were cultured in 12-well plates were placed under a 100× brightfield microscope and filmed using a firmly fixed iPhone 7 plus lens. Each sample was filmed for 10 s at a frame rate of 60 fps. To avoid the effect of temperature on the beating performance, plates were put back to the incubator for 15 min after every three samples were filmed. Original movies were converted to AVI format in order to be processed in the ImageJ software. Then, they were cropped to 512 × 512 pixels, converted to grayscale, and stabilized using the built-in Image Stabilizer plugin.

### M. Beating characterization

CardioMotion plugin was adapted from the open-source MuscleMotion software that was proposed by Sala *et al.*^50^ It is an automated ImageJ tool for the characterization of beating iPSC-CMs using movies. The outcomes of this tool have been proved to resemble those obtained using gold standards. In this study, we used CardioMotion to quantify the contraction amplitude and the peak-to-peak time of the beating iPSC-CMs.

MotionVector plugin is developed in ImageJ for visualizing the local motion of beating iPSC-CMs. It uses a block-matching algorithm as described elsewhere to generate a map of motion vectors.^44^ Here, we set the size of each block (N × N) as 10 × 10 pixels and the maximum searching distance (w) as 4, as shown in Fig. S10 (supplementary material). For each movie, one baseline frame and its neighboring peak frame were extracted and processed using MotionVector plugin. Motion vector maps demonstrate the displacement and the direction of the motion for each block using short line segments [Fig. 7(c) and S11, supplementary material]. Motion aspect ratio is defined as the mean of motion aspect ratios of all blocks. The motion aspect ratio of each block is calculated by dividing the displacement in x direction by that in the y direction.

### N. RNA sequencing

iPSC-CMs were cultured on GelMA substrates for 7 days. The purified iPSC-CMs were lysed with 700 μl QIAZOL (QIAGEN), following the protocol of the miRNeasy Micro Kit with RNase-Free DNase Set (QIAGEN) for RNA digestion. The concentration of eluted RNA is measured with Qubit™ RNA HS Assay Kit, and the RNA integrity number (RIN) is assessed using the Agilent Bioanalyzer RNA6000 Nano. RNA sequencing was conducted by Guangzhou Gene Denovo Biotechnology Co., Ltd. (Guangzhou), and bioinformatic analysis was performed using Omicsmart, a dynamic and interactive online platform for data analysis (https://www.omicsmart.com).

### O. Statistical analysis

All data were expressed as mean ± s.e.m. (standard error of mean). Before conducting significance analysis, outlier data points were removed from each group. This entailed excluding any data points less than *Q*1 − 1.5 × *IQR* or greater than *Q*3 + 1.5 × *IQR* is excluded, where *Q*1 represents the lower quartile, Q3 represents the upper quartile, and *IQR* = *Q*3−*Q*1 is the interquartile range. After removing the outliers, the data undergo normality and homogeneity of variance tests, followed by one-way analysis of variance (ANOVA) for significance analysis. Statistical analysis is performed in R v3.6.3. Bonferroni method was applied to correct p values, which were obtained in multiple pairwise comparisons. Significance level was deemed at p < 0.05 and indicated with an asterisk (^*^) for intra-group comparisons or a number sign (^#^) for inter-group comparisons. All data for statistical analysis were acquired with at least three replications per group.

## SUPPLEMENTARY MATERIAL

See the supplementary material for additional information on micropattern manufacturing (Figs. S1 and S2), the physicochemical properties of GelMA (Figs. S3 and S4), cultivation of iPSC-MCs on micropatterns (Figs. S5 and S6), sarcomere length measurement (Fig S7), motion vector analysis (Figs. S8–S10), and beating and contraction of CMs on the matrix (Movies S1 and S12).

## Data Availability

The data that support the findings of this study are available from the corresponding author upon reasonable request.
